# Supplementary material for: Splice-Junction-Based Mapping of Alternative Isoforms in the Human Proteome
Source: Cell Rep. Author manuscript; Available in PMC 2020 Jan 15. (PMC6961840; doi:10.1016/j.celrep.2019.11.026)
Supplement: 2 [file NIHMS1546469-supplement-2.zip › DF1/PXD010154/Lung/Lung_21_MAP4_MYHDDDLADLVFPSSATADTSIFAGQNDPLKDSYVPLELAK.pdf]

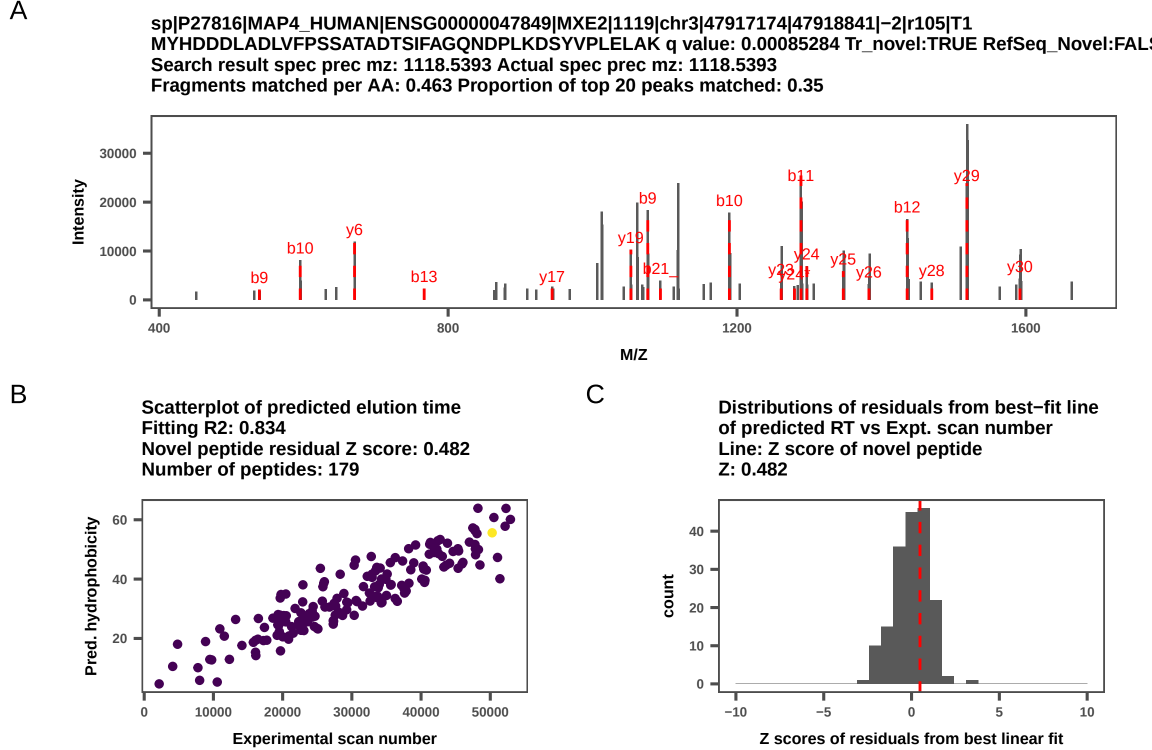
A
sp|P27816|MAP4_HUMAN|ENSG00000047849|MXE2|1119|chr3|47917174|47918841|−2|r105|T1
MYHDDDLADLVFPSSATADTSIFAGQNDPLKDSYVPLELAK q value: 0.00085284 Tr_novel:TRUE RefSeq_Novel:FALS
Search result spec prec mz: 1118.5393 Actual spec prec mz: 1118.5393
Fragments matched per AA: 0.463 Proportion of top 20 peaks matched: 0.35
Intensity
30000
20000
10000
0
b9
b10
y6
b13
y17
y19
b9
b21
b10
y23
b11
y24
y25
y26
b12
y28
y29
y30
400
800
1200
1600
M/Z
B
Scatterplot of predicted elution time
Fitting R2: 0.834
Novel peptide residual Z score: 0.482
Number of peptides: 179
Pred. hydrophobicity
60
40
20
0
10000
20000
30000
40000
50000
Experimental scan number
C
Distributions of residuals from best−fit line
of predicted RT vs Expt. scan number
Line: Z score of novel peptide
Z: 0.482
count
40
30
20
10
0
−10
−5
0
5
10
Z scores of residuals from best linear fit
